# The Impact of Levator Muscle Advancement With and Without Upper Blepharoplasty on Dry-Eye Symptoms in Unilateral Ptosis: A Comparative Study

**DOI:** 10.3390/life15030332

**Published:** 2025-02-21

**Authors:** Dolika D. Vasović, Miodrag Lj. Karamarković, Milan Stojičić, Nikola Musić, Milan Colić, Tanja Kalezić, Jelena Vasilijević, Igor Kovačević, Ivan Marjanović, Miroslav Jeremić, Verica Karamarković, Dejan M. Rašić

**Affiliations:** 1 University Clinical Centre of Serbia, University Eye Hospital, Pasterova 2, 11000 Belgrade, Serbia; 2 Clinic for Burns, Plastic and Reconstructive Surgery, University Clinical Centre of Serbia, 11000 Belgrade, Serbia; 3 Faculty of Medicine, University of Belgrade, 11000 Belgrade, Serbia; 4 Hospital for Plastic, Reconstructive and Aesthetic Surgery “Dr Colić”, 11000 Belgrade, Serbia

**Keywords:** ptosis surgery, blepharoplasty, dry eye, ocular surface health, levator muscle advancement

## Abstract

This study investigates the effects of levator advancement, with and without upper blepharoplasty, on dry-eye symptoms in patients with unilateral ptosis. A total of 92 patients were included, divided into three groups based on surgical intervention: Group A (ptosis correction alone), Group B (ptosis correction with blepharoplasty), and Group C (blepharoplasty alone). Dry-eye parameters were assessed preoperatively and postoperatively at 1, 3, and 6 months using Tear Break-Up Time (TBUT), Schirmer test, corneal and conjunctival staining, and the Ocular Surface Disease Index (OSDI) questionnaire. Our findings indicate that patients in Groups A and B exhibited a temporary increase in dry-eye symptoms, with the most significant effects observed in Group B at the 1- and 3-month follow-ups. By 6 months, dry-eye parameters in all groups returned close to baseline levels, underscoring the reversible nature of these symptoms. This study highlights the importance of preoperative counseling regarding potential temporary dry-eye symptoms, particularly for patients undergoing combined ptosis and blepharoplasty procedures. The results support the safety of these surgical approaches, provided there is appropriate patient monitoring and management to ensure symptom resolution over time.

## 1. Introduction

Ptosis, defined as the pathological drooping of the upper eyelid, can lead to significant functional and aesthetic concerns, often prompting patients to seek surgical intervention. The primary corrective procedure for ptosis is levator muscle advancement and levator repair, particularly suitable for cases where levator muscle dysfunction is the underlying cause. This surgery repositions the eyelid to restore the visual field and improve cosmetic appearance [1]. In some cases, upper blepharoplasty is performed simultaneously to remove excess eyelid skin, further enhancing eyelid contour and achieving optimal aesthetic outcomes [2]. However, combining levator advancement with upper blepharoplasty may have implications for the ocular surface, particularly regarding dry-eye symptoms, a concern that warrants detailed examination [3].

Dry-eye disease is a prevalent and complex disorder of the tear film and ocular surface, characterized by symptoms of discomfort, visual disturbance, and tear instability [4]. Given that eyelid position and function are integral to tear-film distribution and ocular surface protection, surgical alterations of the eyelid may disrupt these dynamics [5]. Previous studies have highlighted that ptosis correction procedures can sometimes exacerbate or induce dry-eye symptoms by modifying blink patterns and eyelid closure [2]. However, the distinct effects of isolated levator muscle advancement versus the combined approach with upper blepharoplasty on dry-eye symptoms remain inadequately explored, particularly in patients with unilateral ptosis, where only one eye undergoes surgical adjustment [5,6].

This study aims to evaluate the impact of levator muscle advancement, with and without concurrent upper blepharoplasty, on dry-eye symptoms in patients with unilateral ptosis, providing insights to help clinicians optimize surgical strategies while addressing the potential risk of postoperative dry-eye symptoms in patients without a prior history of the condition.

## 2. Materials and Methods

This prospective study was designed to include 100 patients scheduled for eyelid surgery, based on sample-size calculations to ensure adequate statistical power (80%) with a significance level of 0.05. Patients were divided into three groups based on the type of surgery: Group A (30 patients undergoing unilateral ptosis correction alone), Group B (35 patients undergoing both ptosis correction and upper blepharoplasty), and Group C (35 patients undergoing upper blepharoplasty alone, without ptosis correction).

All patients were preoperatively screened to ensure they had no prior history of dry-eye symptoms or conditions, allowing us to directly attribute any postoperative changes in dry-eye parameters to the surgical interventions. Exclusion criteria included a history of dry-eye disease, autoimmune or systemic inflammatory conditions, previous eyelid or ocular surgeries, contact lens use, the use of medications affecting tear production, and any ocular surface disease identified upon examination. These criteria ensured that the observed changes in dry-eye symptoms were directly associated with the surgical procedures under study.

The study protocol was approved by the Institutional Review Board, and informed consent was obtained from all participants before enrollment. The research adhered to the principles outlined in the Declaration of Helsinki.

All ptosis correction procedures were performed using the levator muscle advancement technique, which involved creating an incision in the upper eyelid crease, advancing the levator aponeurosis, and securing it with sutures to achieve the desired eyelid position. All patients undergoing the levator muscle advancement technique had fair to good levator function (≥5 mm of excursion). This standardized approach ensured consistency and precision across all procedures. The degree of levator muscle advancement varied depending on ptosis severity and the targeted eyelid position, typically ranging between 2 and 3 mm. As an expected postoperative outcome, temporary lagophthalmos was observed in all cases where ptosis correction was performed, and in most cases, lagophthalmos resolved within 3 months post surgery.

The purpose of upper blepharoplasty in this study was to achieve both functional and aesthetic outcomes. In cases where only blepharoplasty was performed (Group C), no postoperative lagophthalmos was observed. The amount of excess eyelid skin removed was tailored to each patient to optimize results while avoiding adverse ocular surface effects. In cases where blepharoplasty was combined with ptosis correction (Group B), only the strip of skin extending above the eyelid margin was excised, ensuring that the procedure did not contribute to lagophthalmos or ocular surface instability. This individualized approach minimized dry-eye symptoms and maintained optimal eyelid function postoperatively.

A comprehensive dry-eye assessment was conducted at four time points: preoperatively (baseline) and postoperatively at 1, 3, and 6 months. The evaluations included both objective tests and subjective assessments to thoroughly monitor dry-eye status over the study period. To evaluate tear-film stability, we measured Tear Break-Up Time (TBUT) by instilling fluorescein dye into the lower fornix of each eye. After blinking, the interval until the first dry spot appeared on the corneal surface was recorded in seconds. The Schirmer test, used to assess basic tear production, involved placing Schirmer strips in the lower conjunctival sac without topical anesthesia, with the amount of wetting on the strip recorded after five minutes. Corneal and conjunctival staining was evaluated using the Oxford Scheme, a standardized grading system for ocular surface damage, with staining patterns graded from 0 to 5, where higher scores indicated more extensive epithelial disruption. The Meibomian gland function was assessed using a modified grading system based on Arita’s criteria, evaluating various aspects of lid health [7]. This assessment, which was performed using a previously described technique by Mounika et al., enables the morphological and functional evaluation of Meibomian glands [8]. Abnormal lid margin vascularity (ALMV) was graded from 0, indicating normal vascularity, to 3, signifying severe vascularity. Plugging of gland orifices (PGO) was scored from 0, representing no plugging, to 3, which indicated extensive plugging of the gland orifices. Lid margin irregularity (LMI) was graded from 0 (smooth lid margin) to 2 (irregularities present), while Lid margin thickening (LMT) was scored from 0 (no thickening) to 2 (significant thickening). Partial glands (PG) was scored from 0, indicating no partial glands, to 3, reflecting extensive partial gland loss. Gland dropout (GD) was scored from 0 (no dropout) to 2 (substantial gland dropout or loss). Additionally, the Ocular Surface Disease Index (OSDI) questionnaire was administered to all participants before surgery as a baseline measure of dry-eye symptoms and their impact on daily life, as well as at 1, 3, and 6 months postoperatively. The OSDI questionnaire provided a subjective evaluation, with higher scores indicating more severe symptoms. This allowed us to assess patients’ personal experiences of dry eye and monitor changes over the postoperative period across multiple follow-ups.

### Statistical Analysis

Descriptive statistics were used to summarize demographic characteristics among the groups. Statistical analysis was performed using SPSS 26.0 (IBM, Armonk, NY, USA). Due to the ordinal nature of the data, which does not meet parametric assumptions, nonparametric statistical tests were used. To assess differences in dry-eye symptoms among the three groups, the Kruskal–Wallis test was conducted. When significant differences were detected by the Kruskal–Wallis test, post hoc Dunn’s test with Bonferroni correction was employed for pairwise comparisons between groups. Statistical significance was set at *p* < 0.05, with *p* < 0.001 considered highly significant.

## 3. Results

A total of 92 patients were ultimately included in the study, out of the 100 patients initially scheduled for enrollment. Eight patients were excluded due to incomplete follow-up data or withdrawal from the study. The final distribution of patients was as follows: Group A (unilateral ptosis correction alone) included 27 patients, Group B (ptosis correction combined with upper blepharoplasty) included 33 patients, and Group C (upper blepharoplasty alone) included 32 patients.

In Group A, which included patients undergoing ptosis correction only, the mean age was 73.07 ± 7.64 years. Similarly, in Group B (those receiving ptosis correction with upper blepharoplasty), the mean age was 73.24 ± 7.66 years. Group C, consisting of patients who underwent blepharoplasty only, had a slightly higher mean age of 75.59 ± 7.87 years. The gender distribution revealed more notable differences among the groups. In Group A, there was a higher proportion of male patients (66.7%) compared to females (33.3%), and Group B showed a near-balanced distribution with 51.5% males and 48.5% females. In contrast, Group C, the blepharoplasty-only group, had a majority of female patients (65.6%) compared to males (34.4%). These results are presented in Table 1.

Preoperative TBUT values were comparable across all groups, showing no initial differences in tear-film stability (Table 2). At the 1-month postoperative follow-up, TBUT values significantly decreased in Groups A (7 [6; 7]) and B (7 [4.5; 7]) compared to Group C (8 [8; 9]), with a more pronounced reduction in Group B (*p* < 0.001). By 3 months, TBUT showed improvement across all groups, though Groups A and B remained slightly lower than Group C (*p* < 0.05). By the 6-month mark, TBUT values had nearly returned to baseline for all groups, with no significant intergroup differences, indicating recovery of tear-film stability over time (Table 2).

Preoperative tear-production values were consistent among the groups. At 1 and 3 months postoperatively, significant reductions were observed in Groups A and B compared to Group C, with Group B showing the most notable decrease (*p* < 0.001 for both time points). This reduction suggests that ptosis correction and combined surgery temporarily impacted tear production, with combined procedures exerting a stronger effect. By 6 months, Schirmer test values approached baseline in all groups, showing recovery of tear production without significant intergroup differences (Table 2).

Minimal corneal and conjunctival staining scores were recorded preoperatively across all groups, indicating healthy ocular surfaces at baseline. At 1 month postoperatively, staining scores for both corneal and conjunctival staining significantly increased in Groups A (corneal: 3 [1; 3], conjunctival: 3 [2; 3]) and B (corneal: 3 [2; 4], conjunctival: 3 [3; 4]) compared to Group C (corneal: 2 [0; 3], conjunctival: 1 [0; 1]), with Group B showing the most significant increase (*p* < 0.001). By 3 months, staining scores had begun to decrease across all groups, but Groups A and B still showed higher scores than Group C, indicating ongoing but gradually resolving surface changes. At 6 months, although corneal and conjunctival staining scores had approached near-baseline levels, slight yet statistically significant differences persisted between Groups A and B compared to Group C. This suggests that while the ocular surface showed substantial recovery, some alterations due to surgery, particularly in combined procedures, may persist over a longer term.

Preoperative OSDI scores were similar across all groups, indicating no baseline dry-eye symptoms. At 1 and 3 months postoperatively, significant increases were observed in Groups A (20 [19; 22]) and B (22 [18; 23]) compared to Group C (15 [14; 16]), with Group B showing the highest increase (*p* < 0.001). By the 6-month follow-up, OSDI scores for Group A had returned close to baseline, while Group B still displayed slightly elevated scores (*p* < 0.001), suggesting lingering dry-eye symptoms for some patients following combined surgery (Table 2).

MGD parameters, including ALMV, PGO, LMI, LMT, PG, and GD, remained relatively stable across groups throughout the study period. These findings suggest that the surgical interventions had a limited impact on overall Meibomian gland function.

## 4. Discussion

This study provides valuable insights into the effects of ptosis surgery, both alone and in combination with blepharoplasty, on dry-eye symptoms and ocular surface health. While it is well recognized that temporary dry-eye symptoms commonly follow eyelid surgeries [1,3], our study offers a comparative perspective, quantifying the extent and duration of these effects across different surgical procedures. Both surgical interventions significantly impacted tear-film stability, tear production, and ocular surface integrity in the immediate postoperative period, with combined procedures resulting in more pronounced effects. These differences, though subtle, can guide tailored postoperative management and improve outcomes for patients undergoing combined procedures. Importantly, while these changes are largely reversible, our data demonstrate that recovery of dry-eye parameters may take up to six months, consistent with prior studies on ocular surface recovery [9].

The recommendation to minimize tissue trauma and tension during surgery is supported by our findings and aligns with previous research emphasizing technical refinements to reduce postoperative complications, including dry-eye symptoms [4,10]. Although the differences observed across groups may not be dramatic, they hold practical significance for preoperative counseling, particularly in managing patient expectations regarding the temporary nature of dry-eye symptoms and the gradual recovery process. The combined approach of ptosis correction with upper blepharoplasty (Group B) was associated with the most significant short-term exacerbation of dry-eye symptoms, as evidenced by marked reductions in TBUT and Schirmer test values, alongside increased corneal and conjunctival staining scores. These results align with prior research suggesting that surgical manipulation of eyelid tension and position can disrupt normal tear-film dynamics [7,11]. Group B patients exhibited the most pronounced TBUT reduction and heightened staining scores at the 1-month postoperative mark, underscoring the immediate impact on ocular surface integrity. This highlights the need for closer postoperative monitoring and enhanced supportive measures for patients undergoing combined surgeries.

Schirmer test results highlight the temporary disruption of tear production following eyelid surgery. Both ptosis-only (Group A) and combined surgery (Group B) groups experienced reduced tear production at 1 and 3 months postoperatively, with Group B showing the most significant decrease. This temporary reduction likely reflects the impact of surgical manipulation on the lacrimal functional unit, including alterations to eyelid position and blink mechanics. By the 6-month follow-up, Schirmer values across all groups approached baseline, underscoring the reversible nature of tear-production changes over time. These findings are consistent with Hamed Azzam et al. (2024), who reported an initial intensification of dry-eye symptoms post surgery, followed by gradual improvement as eyelid mechanics realigned during the healing process [2].

Corneal and conjunctival staining data similarly highlight the temporary impact of eyelid surgery on the ocular surface [12]. Group B patients exhibited significantly higher staining scores than other groups, particularly at the 1-month follow-up, reflecting increased ocular surface exposure and epithelial disruption. These findings align with Aksu Ceylan et al. (2022), who observed similar effects in patients undergoing combined upper eyelid surgeries [10]. By 3 months, staining scores decreased across all groups, though Groups A and B continued to exhibit higher values than Group C, reflecting a gradual recovery process. At 6 months, staining scores had nearly returned to baseline, with only minor differences persisting, suggesting that the ocular surface is resilient and able to recover fully with time. Our results suggest that combined ptosis and blepharoplasty, despite its temporary disruption, does not lead to permanent damage to the ocular surface, underscoring its safety with careful patient selection.

Patient-reported dry-eye symptoms, measured via OSDI, emphasize the short-term yet reversible impact of eyelid surgery on subjective dryness and visual discomfort. Group B patients reported the highest OSDI scores at 1- and 3-month follow-ups, reflecting the greater disruption caused by combined procedures. Both Groups A and B showed significant early increases in OSDI scores, though symptoms were more severe in Group B, likely due to additional surgical manipulation. By the 6-month follow-up, OSDI scores in Group A had nearly returned to baseline, while Group B scores remained slightly elevated, indicating lingering but diminishing symptoms. These findings align with Zloto et al. (2020), who noted that dry-eye symptoms after combined procedures resolve as tear dynamics stabilize during healing [11].

The Meibomian glands play a crucial role in maintaining tear-film stability by secreting lipids that reduce evaporation. Surgical manipulation of the eyelid during ptosis correction and blepharoplasty can temporarily disrupt gland function due to inflammation, mechanical trauma, or altered eyelid dynamics. In our study, while ALMV showed a slight increase in Group A at 1 month and a mild elevation in Group B at 1 and 3 months, these changes were isolated and did not indicate broader dysfunction in parameters such as PGO or GD. This aligns with findings from Arita et al. (2019), which highlight the resilience of Meibomian gland function despite temporary disruptions caused by surgical trauma and inflammation [7]. These changes are generally reversible as healing progresses and eyelid function normalizes. Similarly, Yesilkaya et al. (2023) observed that temporary impacts on Meibomian gland function after eyelid surgeries typically resolve within the postoperative healing period [13].

The stability of other Meibomian gland parameters in our study underscores the robustness of glandular structures and their ability to recover from surgical intervention. Importantly, the temporary nature of these changes suggests no long-term Meibomian gland dysfunction or permanent tear-film damage. These results also highlight the potential for future research into alternative surgical techniques, such as frontalis suspension or Müller muscle resection, to further minimize disruption to gland function and reduce postoperative dry-eye symptoms. Exploring how different approaches influence eyelid dynamics and tear-film stability could help refine surgical strategies and improve patient outcomes in oculoplastic surgery.

In summary, ptosis surgery, alone or combined with upper blepharoplasty, causes temporary and reversible changes in dry-eye symptoms and ocular surface health. Patients undergoing combined surgery experience more pronounced and prolonged disruptions in parameters such as TBUT, Schirmer values, corneal and conjunctival staining, and OSDI scores. By the 6-month follow-up, most dry-eye parameters returned to baseline or near-baseline levels, emphasizing the reversible nature of these changes and the absence of long-term damage. These findings reinforce the importance of thorough preoperative counseling to prepare patients for transient dry-eye symptoms and appropriate recovery expectations. Counseling is particularly critical for patients undergoing combined procedures, who may require prolonged recovery and closer postoperative monitoring.

## 5. Conclusions

This study demonstrates that ptosis correction, alone or in combination with blepharoplasty, significantly affects dry-eye symptoms in the short term, with combined procedures having a more pronounced and prolonged impact on tear stability, tear production, and ocular surface health. Importantly, these changes are largely reversible, as most parameters return to baseline or near-baseline levels by six months post surgery. While the differences between surgical groups may appear modest, they provide valuable insights into the recovery dynamics of isolated versus combined procedures. These findings reaffirm the safety of ptosis correction and blepharoplasty regarding ocular surface health when appropriate postoperative care is provided.

## Figures and Tables

**Table 1:** Demographic characteristics of study groups by age and gender.

	Group A	Group B	Group C
Age (mean ± SD)	73.07 ± 7.64	73.24 ± 7.66	75.59 ± 7.87
Male (*n*)	18 (66.7%)	17 (51.5%)	11 (34.4%)
Female (*n*)	9 (33.3%)	16 (48.5%)	21 (65.6%)

**Table 2:** Comparison of TBUT, Schirmer test, corneal and conjunctival staining, and OSDI scores among groups at preoperative, 1, 3, and 6 months postoperative. The results are presented as median (Q1; Q3) for each parameter across the three groups: Group A (ptosis correction only), Group B (ptosis correction with blepharoplasty), and Group C (blepharoplasty only). Statistical significance is indicated by symbols: * *p* < 0.05 vs. Group C, ** *p* < 0.001 vs. Group C, # *p* < 0.05 vs. Group B, and ## *p* < 0.001 vs. Group B.

	Group A	Group B	Group C
TBUT			
Preoperative	8 (7; 9)	8 (7; 9)	8 (8; 9)
1 month postoperative	7 (6; 7) **	7 (4.5; 7) **	8 (8; 9)
3 months postoperative	7 (7; 8) *	7 (6; 8) *	8 (8; 9)
6 months postoperative	8 (8; 9)	8 (8; 9)	9 (8; 9)
Schirmer test			
Preoperative	10 (9; 11)	10 (10; 13)	10 (10; 12)
1 month postoperative	7 (7; 8) #, **	6 (5; 7) **	8 (8; 9)
3 months postoperative	8 (7; 8) ##, **	6 (5; 7) **	9 (8; 9)
6 months postoperative	9 (9; 10)	10 (9; 11)	9 (9; 10)
Corneal staining			
Preoperative	1 (0; 2)	1 (0; 1)	1 (0.25; 1)
1 month postoperative	3 (1; 3) *	3 (2; 4) **	2 (0; 3)
3 months postoperative	2 (0; 3) *	2 (0.5; 3) *	1 (0; 2)
6 months postoperative	1 (0; 1) #, **	1 (1; 2) **	0 (0; 1)
Conjunctival staining			
Preoperative	1 (0; 1)	1 (0; 1)	1 (0; 2)
1 month postoperative	3 (2; 3) #, **	3 (3; 4) **	1 (0; 1)
3 months postoperative	1 (1; 2) **	2 (1; 2.5) **	0 (0; 1)
6 months postoperative	1 (0; 1) *	1 (0.5; 2) *	0.5 (0; 1)
OSDI			
Preoperative	13 (10; 15)	13 (10; 15)	12 (10; 14)
1 month postoperative	20 (19; 22) **	22 (18; 23) **	15 (14; 16)
3 months postoperative	19 (15; 19) **	17 (16; 21) **	14 (12; 16)
6 months postoperative	12 (10; 15) ##, **	16 (14; 18) **	13 (11.25; 15)

## Data Availability

Data are contained within the article.
